# Genetic diversity among two native Indian chicken populations using *cytochrome c oxidase subunit* I and *cytochrome b* DNA barcodes

**DOI:** 10.14202/vetworld.2021.1389-1397

**Published:** 2021-05-30

**Authors:** Ankit R. Dave, Dhaval F. Chaudhary, Pooja M. Mankad, Prakash G. Koringa, D. N. Rank

**Affiliations:** 1 Department of Animal Genetics and Breeding, College of Veterinary Science and Animal Husbandry, Anand, Anand Agricultural University, Gujarat, India; 2 Department of Animal Biotechnology, College of Veterinary Science and Animal Husbandry, Anand, Anand Agricultural University, Gujarat, India

**Keywords:** *Cytochrome b*, *Cytochrome oxidase*, genetic diversity, mitochondrial DNA, phylogenetics, poultry

## Abstract

**Background and Aim:**

India has large varieties (recognized, unrecognized) of native chickens (Desi) scattered throughout the country, managed under scavenging system different from commercial chicken breeds. However, they are less investigated for genetic diversity they harbor. The present study was planned to evaluate genetic diversity among two native chicken populations of North Gujarat (proposed Aravali breed) and South Gujarat (Ankleshwar breed). Aravali chicken, a distinct population with unique characters different from the registered chicken breeds of India is under process to be registered as a new chicken breed of Gujarat, India.

**Materials and Methods:**

Two mitochondrial markers, namely*, cytochrome oxidase c subunit I* (COX I) and *cytochrome b* (Cyt b) genes were studied across 10 birds from each population. Methodology included sample collection (blood), DNA isolation (manual), polymerase chain reaction amplification of mitochondrial genes, Sanger sequencing, and purification followed by data analysis using various softwares.

**Results:**

Haplotype analysis of the COX I gene unveiled a total eight and three haplotypes from the Aravali and Ankleshwar populations, respectively, with haplotype diversity (Hd) of 92.70 % for the Aravali and 34.50% for the Ankleshwar breed. Haplotype analysis of the Cyt b gene revealed a total of four haplotypes from the Aravali population with 60% Hd and no polymorphism in Ankleshwar breed. The phylogenetic analysis uncovered Red Jungle Fowl and Gray Jungle Fowl as prime roots for both populations and all domestic chicken breeds.

**Conclusion:**

Study findings indicated high genetic variability in Aravali chicken populations with COX I mitochondrial marker being more informative for evaluating genetic diversity in chickens.

## Introduction

Biodiversity is the variation of life on Earth that is most important for several healthy ecosystems. Species diversity and genetic diversity within species play a key role in stable functioning of ecosystems. Genetic diversity is the variation of alleles and genotypes within the genome [1]. Genetic diversity among the domestic livestock is commonly measured through molecular markers such as microsatellites, mitochondrial DNA sequences, single-nucleotide polymorphisms (SNPs) available on commercial chips, and the complete metagenome sequences. Mitochondria are the powerhouse of a cell that produces energy for the cell. The animal mtDNA is ~ 17 kb of circular, coiled, multiple copied, extra-nuclear genome with high mutation rate and evolution rate of about 5-10 times faster than nuclear DNA, making it highly diverse within a species and fundamental material for phylogenetic and genetic diversity studies [2]. It has 37 genes, 13 protein-coding, two ribosomal RNA, and 22 transfer RNA genes [3], a mong which *cytochrome* b (Cyt b) and *cytochrome c oxidase subunit* I (COX I) are important for phylogenetic and genetic diversity studies.

Chicken is an iconic model to study domestic birds which have enormous population with hundreds of breeds and strains that can be grown in a controlled environment. A huge chicken population of 851.81 million with tremendous growth in chicken industry contributes significantly to the Indian economy (Anonymous, 2019). A total of 20 chicken breeds are registered in India (NBAGR, 2019) to which Gujarat contributes Ankleshwar and Busra. Indigenous chicken plays an important role in rural and/or tribal areas due to the number of different qualities, namely, disease resistance, efficient adaptability-survivability-mothering ability, and natural scavenging-nesting habit [4]. However, tribal areas of the eastern fringe of Gujarat have a poor reach of technologies and are less explored with respect to animal genetic resources. Moreover, the ancestors of the domestic chicken (*Gallus gallus domesticus*) are two wild avian species, namely, Red Jungle Fowl (*G. gallus*) and Gray Jungle Fowl (*Gallus sonneratii*) [5]. Domestication changes several traits in wild ancestor species. Hence, well-planned scientific study needs to be focused on such aspects.

Hence, the molecular investigation was undertaken for the determination of genetic diversity of unique and native chicken populations of North (proposed Aravali breed) as well as South Gujarat (Ankleshwar breed) through mitochondrial markers (COX I; and Cyt b).

## Materials and Methods

### Ethical approval

This study was approved by the Institutional Animal Ethics Committee of College of Veterinary Science and Animal Husbandry, Anand Agricultural University, Anand, Gujarat, India.

### Study period and location

The study was carried out from October 2018 to September 2019. The sampling was conducted from October to November 2018 in Aravali, Banaskantha, Sabarkantha and Bharuch district of Gujarat, India. The wet laboratory research work was done January to September 2019 at the Department of Animal Genetics and Breeding, Anand Agricultural University, Anand, Gujarat, India.

### Sample collection and DNA isolation

The present study included two native populations of Aravali and Ankleshwar chicken (10 birds from each population). For sampling, we selected 10-12 villages from core breeding tracts of phenotypically “true to breed” of both populations. The blood samples were collected from Aravali (3-5 birds/village) and Ankleshwar (5-10 birds/village) breeds reared as backyard poultry farming across three districts Aravali, Banaskantha, and Sabarkantha of the North Gujarat (Aravali) and Bharuch district of South Gujarat (Ankleshwar). DNA extractions from whole blood samples were carried out using manual method [6]. Quality and purity of DNA were checked by agarose gel electrophoresis (0.8%) at 80 V for 60 min. Quality and concentration of DNA were also checked by ND-1000 Spectrophotometer (NanoDrop Technologies, Inc., USA).

### Polymerase chain reaction (PCR) amplification of mitochondrial genes

PCR amplification was carried out using two primer pairs, namely, BirdF1 and COIbirdR2 for COX I [7]; L15662 and H16065 for Cyt b [8]. PCR amplification was carried out in a total reaction volume of 25 mL using ×2 PCR Master Mix (Emerald, TaKara, Japan) primers and 50-100 ng DNA on 2720 thermal cycler (Applied Biosystems, Massachusetts, USA). PCR cycling conditions for the amplification of COX I mitochondrial gene consisted 94°C for 1 min, followed by five cycles (at 94°C for 1 min, at 45°C for 30 s, at 72°C for 1 min), 35 cycles (at 94°C for 1 min, at 54°C for 30 s, at 72°C for 1 min), and a final extension step at 72°C for 5 min. PCR cycling conditions for the amplification of Cyt b mitochondrial gene consisted 96ºC for 5 min, followed by 40 cycles of 95°C for 45 s, at 56°C for 1 min, at 72°C for 1 min, and a final extension step at 72°C for 10 min. PCR amplification was confirmed by agarose gel electrophoresis (2 %) with 100 bp DNA ladder (Thermo Scientific, USA) at 80 V for 45-60 min. The amplified products were visualized as compact bands of the expected size (~ 746 bp for COX I and ~ 415 bp for Cyt b) under ultraviolet light and documented by gel documentation system (Syngene, Gene Genius Bio-Imaging, USA). All PCR products were purified using a QIAquick gel extraction kit to obtain accurate sequence information.

### Sequencing, purification, and data analysis

Cycle sequencing of all samples was carried out in a total reaction volume of 20 μL using BigDye^®^ Terminator v3.1 Cycle Sequencing Ready Reaction-100 mix (Thermo Fisher Scientific, Applied Biosystems), BigDye^®^ Terminator v1.1 and v3.1 ×5 Sequencing Buffer (Thermo Fisher Scientific, Applied Biosystems), forward/reverse primers, and 50-70 ng/μL purified PCR product (Gel extracted DNA) on 2720 thermal cycler (Applied Biosystems). Cycle sequencing conditions consisted 95°C for 5 min, followed by 32 cycles of 95°C for 20 s, at 55°C for 15 s, and at 60°C for 4 min. All sequenced reactions were purified using Zymo Research DNA Sequencing Clean-upTM Kit (The Epigenetics Company, USA) and sequenced by capillary electrophoresis on an automated DNA sequencer (ABI PRISM 3500 Genetic Analyzer). All the raw sequences were curated and assembled using bioinformatics tools, namely, Sequencing Analysis 5.2 (Thermo Fisher Scientific, Applied Biosystem, India) and Clone Manager Suite 9 (Sci Ed Software, Westminster, Colorado, USA). All the consensus sequences were then aligned and trimmed using bioinformatics software, namely, CLUSTALW and BioEdit Sequence Alignment Editor for the haplotyping analysis. The haplotyping was done using bioinformatics software DnaSP v6.12.03 (University of Barcelona, Spain) (DNA Sequence Polymorphism), considering *G. gallus* as reference.

## Results

### PCR amplification of mitochondrial genes

**Figure-1:**
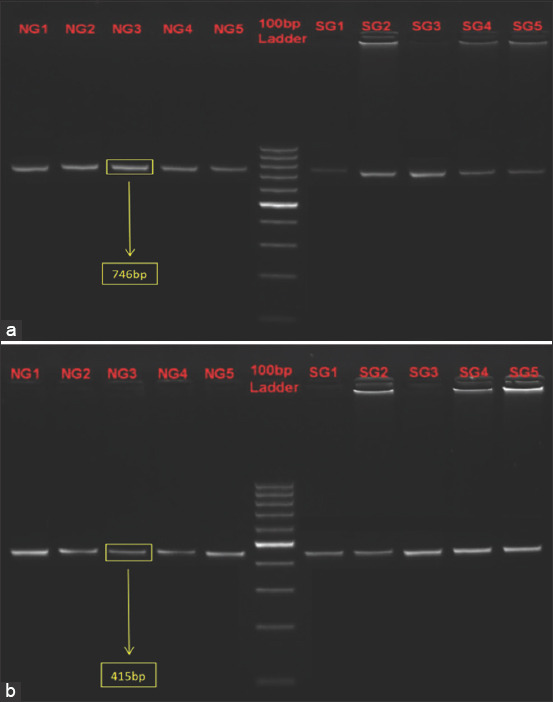
Agarose gel electrophoresis (2%) of COX I (746 bp, a) and Cyt b (415 bp, b) amplicons of representative samples of Aravali (lanes: 1-5, in a and b) and Ankleshwar (lanes: 7-11, in a and b) populations. Size standard: 100 bp ladder (lane 6 in a and b).

DNA barcoding of 20 samples (10 each from Aravali and Ankleshwar breed) was done successfully by PCR amplification of COX I (~ 746 bp) and Cyt b (~ 415 bp) gene fragments and sequencing. Representative image of amplified PCR product is shown in Figure-1.

### Sequencing and data analysis

**Figure-2:**
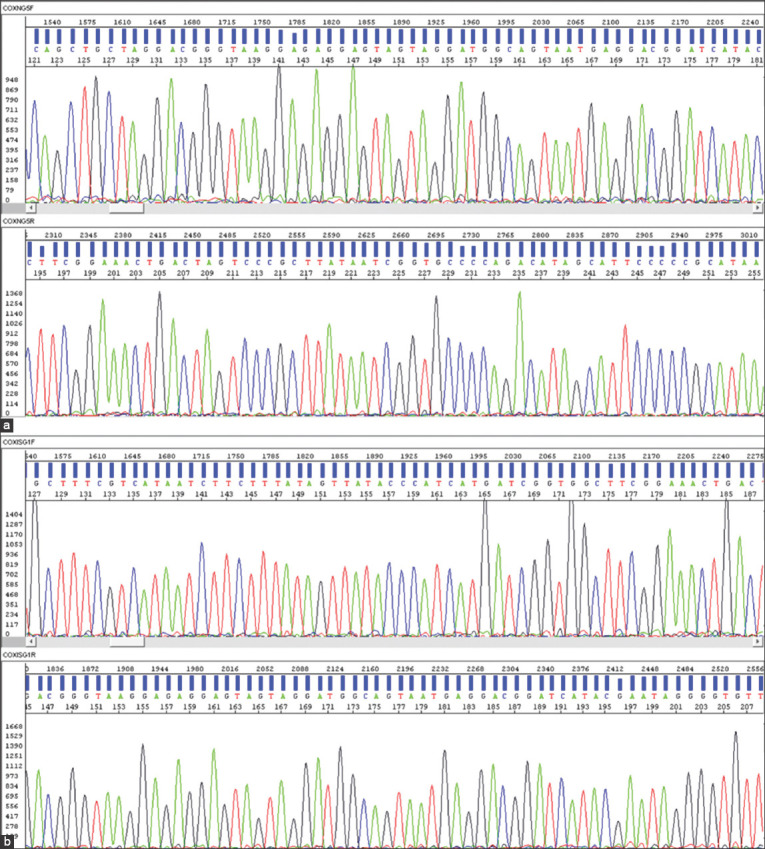
Raw sequences (blue bars) and electropherogram (colored peaks) of COX I gene of representative samples of Aravali (a) and Ankleshwar (b) population. (a) NG05 (b) SG01.

**Figure-3:**
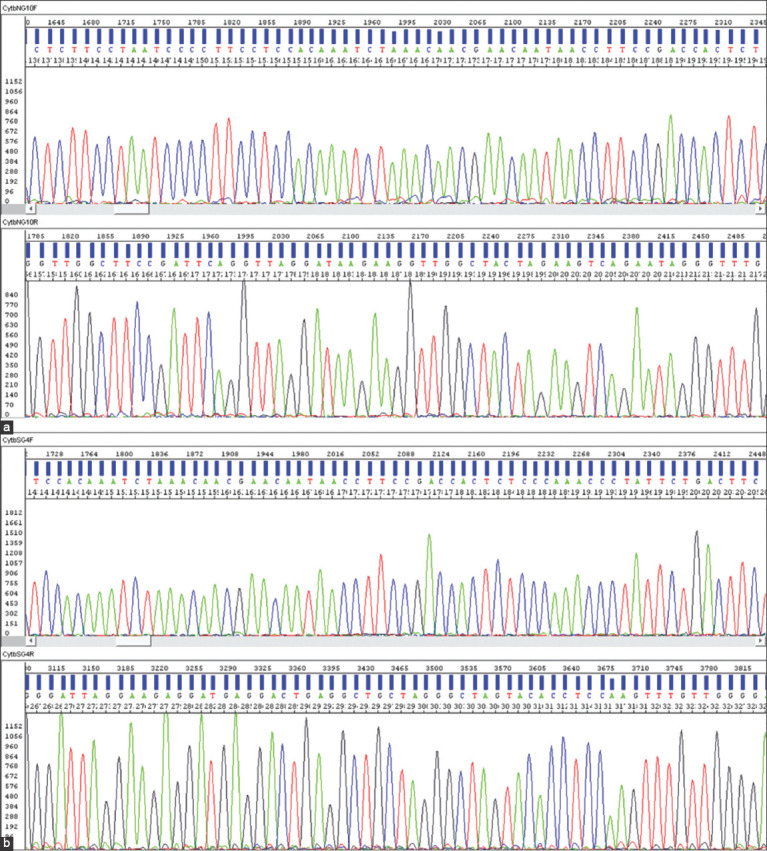
Raw sequences (blue bars) and electropherogram (colored peaks) of Cyt b gene of representative samples of Aravali (a) and Ankleshwar (b) population. (a) NG10 (b) SG04.

Representative electropherogram images of COX I and Cyt b raw gene sequences are shown in Figures-2 and 3, respectively. Consensus sequences of COX I gene (608 bp to 756 bp) and Cyt b gene (378 bp to 496 bp) were subjected to BLASTN at NCBI (http://www.ncbi.nlm.nih.gov/blast). All the consensus sequences were matched with the complete mitochondrial genome of *G. gallus*. A complete mitochondrial genomes of Red Jungle Fowl (*Gallus gallus murghi*; GU261708.1) and Gray Jungle Fowl (*G. sonneratii*; AP003320.1) were downloaded from NCBI site.

Comparative haplotype analysis between Aravali and Ankleshwar chicken populations is shown in Table-1. Furthermore, the molecular definitions of haplotypes identified are shown in Tables-2-4.

**Table-1:** Comparative haplotype analysis of COX I and Cyt b genes between Aravali and Ankleshwar chicken populations.

Parameter/chicken population	COX I gene		Cyt b gene	
	
	Aravali	Ankleshwar	Aravali	Ankleshwar
Selected region/number of sites	1–653	1–682	1–345	1–327
Number of total sites (excluding gaps/missing data)	569	647	345	327
Number of polymorphic sites (S)	12	03	03	-
Number of haplotype (h)	08	03	04	-
Haplotype/gene diversity (Hd)	0.927	0.345	0.600	-
Variance of Hd	0.00442	0.02967	0.02369	-
Standard deviation of Hd	0.066	0.172	0.154	-
Nucleotide diversity (Pi/π) Jukes and Cantor	0.22857	0.00084	0.00233	-
Average number of nucleotide difference (k)	3.6	0.545	0.800	-

**Table-2:** Molecular definitions of eight COX I haplotype from Aravali chicken population.

Polymorphic site location	45	46	353	355	368	418	453	484	508	520	624	632
Reference sequence *Gallus gallus*	G	C	A	T	A	A	C	T	C	G	G	G
NGXHp_1 (NG 1, 3)	-	A	-	C	G	-	A	G	A	T	A	C
NGXHp_2 (NG 2)	A	G	C	G	T	-	A	G	A	C	-	C
NGXHp_3 (NG 4)	-	A	-	C	G	-	G	A	A	C	-	C
NGXHp_4 (NG 5)	-	A	-	C	G	-	A	G	A	C	A	C
NGXHp_5 (NG 6)	-	A	-	C	G	-	G	G	A	C	-	C
NGXHp_6 (NG 7, 8)	-	A	-	C	G	-	A	G	A	C	A	C
NGXHp_7 (NG 9)	-	A	-	C	G	T	A	G	T	T	A	-
NGXHp_8 (NG 10)	-	A	-	C	G	-	G	A	A	C	-	A

**Table-3:** Molecular definition of three COX I haplotypes from Ankleshwar chicken population.

Polymorphic site location	29	37	79
Reference sequence *Gallus gallus*	C	C	C
SGXHp_1 (SG 1, 4-10)	-	T	T
SGXHp_2 (SG 2)	A	T	T
SGXHp_3 (SG 3)	G	T	T

**Table-4:** Molecular definition of four Cyt b haplotypes from Aravali chicken population.

Polymorphic site location	6	28	46
Reference sequence *Gallus gallus*	T	C	C
NGbHp_1 (NG 1)	C	-	G
NGbHp_2 (NG 2)	-	-	G
NGbHp_3 (NG 3-9)	C	-	-

### Haplotype analysis for COX I gene barcodes

Total numbers of polymorphic sites were higher (12) in Aravali chicken population than in Ankleshwar chicken population (three). The total numbers of haplotypes found were also higher in Aravali chicken population (eight haplotypes with haplotype diversity [Hd] 92.70%) than Ankleshwar chicken population (three haplotypes with Hd 34.50%). In the Aravali population, except haplotypes NGXHp_1 and NGXHp_6, all other haplotypes were represented by single samples, whereas Ankleshwar chicken population had 80% frequency of the haplotype SGXHp_1 with other two haplotypes, namely, SGXHp_2 and SGXHp_3 represented by single samples. The nucleotide diversity in Aravali chicken population was significantly higher (22.85%) than Ankleshwar chicken population (0.08%). It is pertinent to mention that consensus sequence length was slightly shorter (653 bp) in Aravali than Ankleshwar (682 bp) chicken populations.

### Haplotype analysis for Cyt b gene barcodes

After quality filtering, overall selected sequence length was 345 bp for Aravali chicken population and 327 bp for Ankleshwar chicken population. Total numbers of polymorphic sites in Aravali chicken population were three with 0.23% nucleotide diversity and total four haplotypes were found with Hd 60%, whereas Ankleshwar chicken population had 100% identical sequences, which indicated that the Aravali chicken population had higher genetic diversity compared to the Ankleshwar chicken population (with more number of haplotypes and higher haplotype as well as nucleotide diversity).

### The phylogenetic analysis

**Figure-4:**
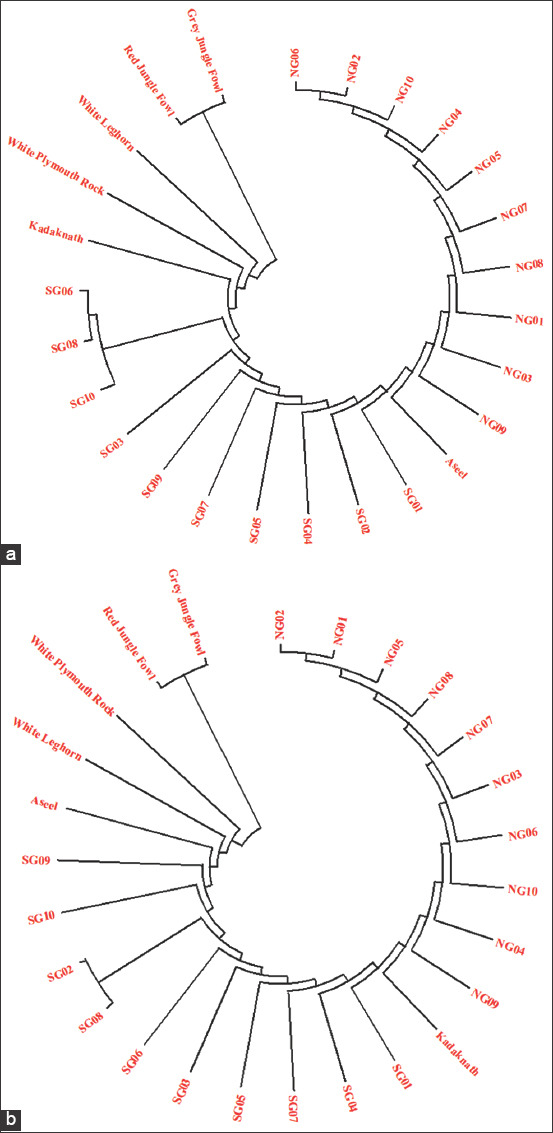
Phylogenetic trees made from obtained sequences of COX I gene (a), Cyt b gene (b) of Aravali and Ankleshwar chicken populations with sequences of domestic chicken breeds and two wild ancestors (Red Jungle Fowl and Gray Jungle Fowl). Aravali and Ankleshwar chicken show divergence from classical layer (White Leghorn) and meat (White Plymouth Rock).

The phylogenetic analysis was performed using bioinformatics software, namely, MEGA-X v.10.0.5 (Pennsylvania State University, Pennsylvania, USA) (Molecular Evolutionary Genetics Analysis). For comparative phylogeny, COX I and Cyt b gene sequences of various domestic chicken breeds were downloaded from the NCBI site (http://www.ncbi.nlm.nih.gov), namely, Aseel (KP211418.1), Kadaknath (KP211425.1), White Leghorn (AP003317.1), White Plymouth Rock (AP003318.1), Minorca (AF354171.1), and wild ancestor of domestic chicken breeds, namely, Red Jungle Fowl (*Gallus* *gallus*; GU261708.1) and Gray Jungle Fowl (*G. sonneratii*; AP003320.1). Phylogenetic trees were constructed using Neighbor-Joining method (1000 bootstrap replications; Kimura 2-parameter model) and presented in Figure-4. The present findings supported that the Red Jungle Fowl and Gray Jungle Fowl form prime roots for all the existing domestic chicken breeds. Furthermore, analysis suggested that Ankleshwar chicken population might have evolved earlier than Aravali chicken population.

### Bold sequence accession numbers

All sequences were submitted in BOLD SYSTEMS (Barcode of Life Data System; www.boldsystems.org) under the project name “ARD.” The unique BOLD sequence IDs for respective samples are indicated in Table-5.

**Table-5:** Unique BOLD sequence IDs of submitted sequences in BOLD SYSTEMS for respective samples.

S. No.	Chicken breed	Institutional sample ID	BOLD sample ID	BOLD sequence ID	

				COX I gene	Cyt b gene
1.	North Gujarat/Aravali	NG01	PB_NG01	ARD001-19.COI-5P	ARD001-19.CYTB
2.		NG02	PB_NG02	ARD002-19.COI-5P	ARD002-19.CYTB
3.		NG03	PB_NG03	ARD003-19.COI-5P	ARD003-19.CYTB
4.		NG04	PB_NG04	ARD004-19.COI-5P	ARD004-19.CYTB
5.		NG05	PB_NG05	ARD005-19.COI-5P	ARD005-19.CYTB
6.		NG06	PB_NG06	ARD006-19.COI-5P	ARD006-19.CYTB
7.		NG07	PB_NG07	ARD007-19.COI-5P	ARD007-19.CYTB
8.		NG08	PB_NG08	ARD008-19.COI-5P	ARD008-19.CYTB
9.		NG09	PB_NG09	ARD009-19.COI-5P	ARD009-19.CYTB
10.		NG10	PB_NG10	ARD010-19.COI-5P	ARD010-19.CYTB
11.	South Gujarat/Ankleshwar	SG01	PB_SG01	ARD011-19.COI-5P	ARD011-19.CYTB
12.		SG02	PB_SG02	ARD012-19.COI-5P	ARD012-19.CYTB
13.		SG03	PB_SG03	ARD013-19.COI-5P	ARD013-19.CYTB
14.		SG04	PB_SG04	ARD014-19.COI-5P	ARD014-19.CYTB
15.		SG05	PB_SG05	ARD015-19.COI-5P	ARD015-19.CYTB
16.		SG06	PB_SG06	ARD016-19.COI-5P	ARD016-19.CYTB
17.		SG07	PB_SG07	ARD017-19.COI-5P	ARD017-19.CYTB
18.		SG08	PB_SG08	ARD018-19.COI-5P	ARD018-19.CYTB
19.		SG09	PB_SG09	ARD019-19.COI-5P	ARD019-19.CYTB
20.		SG10	PB_SG10	ARD020-19.COI-5P	ARD020-19.CYTB

## Discussion

Originally 60 samples each from both breeds were collected from their respective breeding tracts and evaluated for genetic diversity using microsatellite markers (unpublished data). Both the breeds are reared by tribal community around 500 kilometers apart. Aravali is sparsely distributed across three districts (Aravali, Banaskantha, and Sabarkantha, approximately 10,000 square kilometers) of North Gujarat; while Ankleshwar is more concentrated in and around Ankleshwar town and is sparsely distributed in forested area in approximately 5000 square kilometers of Bharuch district of South Gujarat. Aravali is mostly reared in the group of 5-10 birds (including 1-2 males) per household, while Ankleshwar is reared in and around Ankleshwar town in the groups of 10-20 birds including 2-4 males and in a smaller group in surrounding areas. Accordingly, sampling was done from 10 to 12 villages of core breeding tracts, with phenotypically true to breed populations of both chicken breeds, 3-5 birds per villages for Aravali and 5-10 birds per villages for Ankleshwar. The 10×2 samples were the subset of original sample set. Sex ratio was 3 males:7 females.

Hd and nucleotide diversity (Pi) of populations are the main indexes for evaluating mtDNA variation and genetic diversity of a breed or a population. COX I and Cyt b haplotype analysis in this study revealed Aravali population to be genetically more variable harboring more polymorphic sites (12 for COX I and three for Cyt b) and haplotypes (eight for COX I and four for Cyt b) with higher haplotype (0.927 for COX I and 0.600 for Cyt b) and nucleotide diversity (0.228 for COX I and 0.0023 for Cyt b) than Ankleshwar breed. This supports our findings that Aravali are genetically more variable than Ankleshwar based on microsatellite profiling (unpublished data).

These markers (COX I and Cyt b) have been used by number of investigators to identify native chicken, to study genetic diversity, population structure, evolution, and origin of native chicken breeds. High genetic diversity as revealed by a higher number of polymorphic sites defining more number of haplotypes was reported in Chinese black bone chickens (22 and 24) [9], Chinese native chickens (5 and 10) [10], Tibetan chicken (4 and 6) [11], Chinese native chickens (24 and 24) [12] in COX I marker and in black boned chicken breeds (17 and 8) [13], and game chicken breed of China (7 and 6) [14] in Cyt b marker. However, these markers (COX I and Cyt b) are more commonly used in evaluation of genetic diversity among wild populations than domesticated populations. High genetic diversity with the higher number of polymorphic sites defining more number of haplotypes was reported in Mediterranean breeding colonies of Greater Flamingo (*Phoenicopterus roseus*) (15 and 16) [15] and Lesser Flamingos (*Phoenicopterus minor*) from Africa and Gujarat (11 and 14) [16]. Although, this is a small study including only two breeds, it supported the diversity estimation by microsatellite markers (data not shown).

Other mitochondrial markers such as D-loop (control region) have also been used by various investigators to explore diversity among different native chicken breeds across the world. Higher number of polymorphic sites defining more number of haplotypes were reported in Samar Philippines native chickens (17 and 5) [17], Hungarian native chickens (17 and 11) [18], Egyptian native chickens (28 and 18) [19], native chicken breeds of Jiangsu (33 and 19) [20], and chicken breeds of Korea (84 and 31) [21]. These studies showed high genetic diversity with higher haplotype and nucleotide diversity in Samar Philippines native chickens (0.92 and 0.0056) [22], Hungarian native chickens (0.626 and 0.0049) [18], Egyptian native chickens (0.81 and 0.0045) [19], native chicken breeds of Jiangsu (0.862 and 0.00591) [20], and chicken breeds of Korea (0.604 and 0.007) [21].

Today, newer markers such as SNPs and whole mitogenome sequencing are available for assessing the genetic diversity among domestic breeds. However, considering the costs involved and assess to technology, barcoding markers were used in the present study. SNPs have been used for genome-wide analysis to assess the conservation status and the genomic variability of Italian chicken breeds [22] as well as to understand genetic structures of these (indigenous, commercial, gamecock, and wild ancestral chicken) breeds under different selection pressure [23]. Furthermore, mitogenome sequences have been utilized to assess hereditary divergence between 22 Asian native breeds and seven native Indian chicken breeds [24], to investigate phylogenetic evolution and genetic diversity of Tibetan chicken [25], Tosa-Jidori of Japan [26], Huainan Partridge [27], and Huangshan Black [28] chickens of China. However, microsatellites and DNA barcodes still accepted as markers of choice because they are technically less demanding and cost-effective in evaluation. Ample literature is available on use of microsatellite markers in domestic chicken [29-31]. Nonetheless, uses of SNP markers and whole mitogenome sequencing have recently been appeared in the publication, which can be used to evaluate genetic diversity of domestic chicken.

## Conclusion

As far as the authors are aware, this is the first diversity study involving mitochondrial markers in domestic chicken in India. Aravali chicken population seems to be more genetically diverse, as reflected by higher number of COX I and Cyt b haplotypes than the Ankleshwar chicken population. Out of two mitochondrial markers, COX I stood out to be more informative than Cyt b for the use of genetic diversity study. Considering limited power of these markers, recent markers such as SNPs and whole mitogenome sequencing are suggested to evaluate genetic diversity of chicken with ideal sampling strategies involving more number of male samples.

## Authors’ Contributions

ARD, PGK, and DNR: Designed the study. ARD and DFC: Collected the samples. ARD and PMM: Carried out research in the laboratory. ARD: Analyzed, wrote, and revised the manuscripts. DNR and PGK: Supervised the manuscript. All authors have read and approved the final manuscript.
